# Involvement of an Arginine Triplet in M1 Matrix Protein Interaction with Membranes and in M1 Recruitment into Virus-Like Particles of the Influenza A(H1N1)pdm09 Virus

**DOI:** 10.1371/journal.pone.0165421

**Published:** 2016-11-04

**Authors:** Adeline Kerviel, Shantoshini Dash, Olivier Moncorgé, Baptiste Panthu, Jan Prchal, Didier Décimo, Théophile Ohlmann, Bruno Lina, Cyril Favard, Etienne Decroly, Michèle Ottmann, Philippe Roingeard, Delphine Muriaux

**Affiliations:** 1 Centre d'études d'agents Pathogènes et Biotechnologies pour la Santé (CPBS), CNRS & Université of Montpellier, Montpellier, France; 2 CIRI, INSERM U 1111, France & ENS de Lyon, Lyon, France; 3 Université de Lyon, Université Lyon 1, Faculté de Médecine Lyon Est, Laboratoire de Virologie et Pathologie Humaine, EA 4610, Lyon, France; 4 INSERM U966, Université François Rabelais & CHRU de Tours, Tours, France; 5 Aix-Marseille Université & CNRS, AFMB UMR 7257, 163 Avenue de Luminy, 13288 Marseille cedex 09, France; Johns Hopkins University Bloomberg School of Public Health, UNITED STATES

## Abstract

The influenza A(H1N1)pdm09 virus caused the first influenza pandemic of the 21st century. In this study, we wanted to decipher the role of conserved basic residues of the viral M1 matrix protein in virus assembly and release. M1 plays many roles in the influenza virus replication cycle. Specifically, it participates in viral particle assembly, can associate with the viral ribonucleoprotein complexes and can bind to the cell plasma membrane and/or the cytoplasmic tail of viral transmembrane proteins. M1 contains an N-terminal domain of 164 amino acids with two basic domains: the nuclear localization signal on helix 6 and an arginine triplet (R76/77/78) on helix 5. To investigate the role of these two M1 basic domains in influenza A(H1N1)pdm09 virus molecular assembly, we analyzed M1 attachment to membranes, virus-like particle (VLP) production and virus infectivity. *In vitro*, M1 binding to large unilamellar vesicles (LUVs), which contain negatively charged lipids, decreased significantly when the M1 R76/77/78 motif was mutated. In cells, M1 alone was mainly observed in the nucleus (47%) and in the cytosol (42%). Conversely, when co-expressed with the viral proteins NS1/NEP and M2, M1 was relocated to the cell membranes (55%), as shown by subcellular fractionation experiments. This minimal system allowed the production of M1 containing-VLPs. However, M1 with mutations in the arginine triplet accumulated in intracellular clusters and its incorporation in VLPs was strongly diminished. M2 over-expression was essential for M1 membrane localization and VLP production, whereas the viral trans-membrane proteins HA and NA seemed dispensable. These results suggest that the M1 arginine triplet participates in M1 interaction with membranes. This R76/77/78 motif is essential for M1 incorporation in virus particles and the importance of this motif was confirmed by reverse genetic demonstrating that its mutation is lethal for the virus. These results highlight the molecular mechanism of M1-membrane interaction during the formation of influenza A(H1N1)pdm09 virus particles which is essential for infectivity.

## 1-Introduction

The influenza A(H1N1)pdm09 strain spread in 2009 and caused the first influenza pandemic of the 21^st^ century. The influenza A(H1N1)pdm virus represents a public health threat and is still circulating in humans. A better understanding of its replication cycle and viral transmission is crucial for developping new antiviral strategies which might help to control the next pandemics. Influenza viruses belong to the *Orthomyxoviridae* family of negative-sense, single-stranded and segmented RNA genome viruses. The influenza A virus is composed of eight viral RNA segments (PB2, PB1, PA, HA, NP, NA, M and NS) that encode ten major proteins. The production of new infectious virions requires their simultaneous incorporation during virus assembly. Assembly and budding of influenza virions is a multi-step process that occurs at the cell plasma membrane of infected cells [1]. Indeed, influenza viruses have a lipid membrane that is derived from the host cell and that harbors the viral transmembrane proteins HA and NA and some M2, the viral ion channel protein. During the early steps of the replication cycle, M2 is involved in virus uncoating and during the late steps in promoting the scission of newly formed particles via an endosomal sorting complexes required for transcription (ESCRT)-independent process [2]. The virus "core" includes the eight viral ribonucleoprotein (vRNP) complexes each of which is composed of one viral RNA segment that encodes one or more viral proteins coated by nucleoproteins (NP). This “core” is complexed with a polymerase complex made of three subunits (PB1, PB2, and PA). The nuclear export protein NEP (also known as NS2) also is found in virions [3] and few copies of Non Structural protein 1 (NS1) can also be detected in viral particles [4]. The matrix protein M1, the most abundant protein in viral particles, is localized underneath the viral envelope between the host cell membrane and the vRNPs or the transmembrane viral proteins and the vRNPs. M1 has a central role in the assembly and release of viral particles, as indicated by the finding that both processes are abrogated in its absence [5].

**Fig 1:**
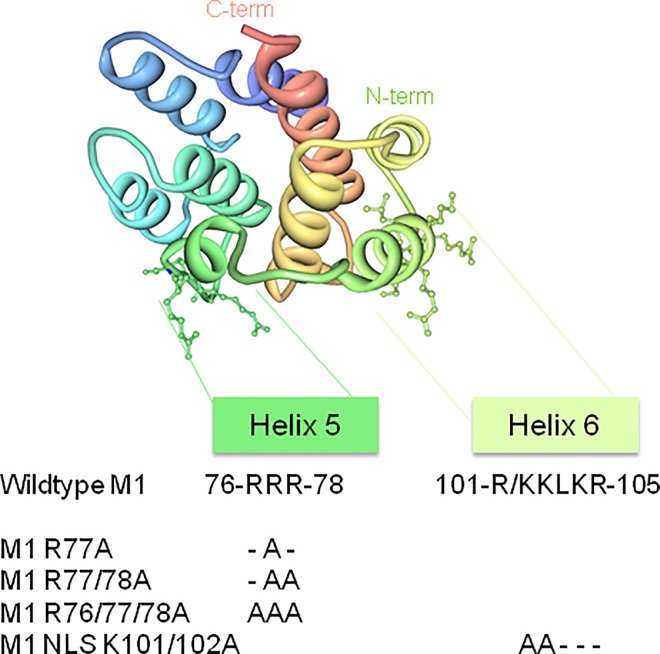
The N-terminal M1 viral protein and its basic mutants. Schematic representation of the N-terminal domain of the influenza A M1 protein obtained using the I-TASSER software (http://zhanglab.ccmb.med.umich.edu/I-TASSER/, [45]*)*. Helix 5 and helix 6 are shown to indicate the position of the two N-terminal basic R76/77/78 (Arginine triplet) and K101/102 (NLS) motifs of M1. The respective M1 mutants obtained by directed-site mutagenesis are shown.

Upon influenza virus assembly, M1 and the vRNPs must reach the plasma membrane (the site of viral assembly) and interact with the glycoproteins HA and NA. M1 can associate with HA and NA during their traffic to the apical membrane microdomains *via* the exocytic pathway [6] [7]. M1-vRNP complexes can also use the cytoskeleton to reach the virus assembly sites through NP-cytoskeleton interactions [8] [9]. Alternatively, M1-vRNP complexes can use the recycling endosomal pathway, via RAB11 interactions, for targeting the cell membrane [10]. However, it is not well established how M1 is involved in assembly site recognition at the cell membrane. Indeed, virus assembly and budding occur at the plasma membrane and a lipidomic study has shown that virions are enriched in cholesterol and sphingolipids [11]. The association of HA and NA with lipid rafts is essential for virus replication, but M2 seems to be excluded from lipid rafts [12]. It has been proposed that M2 binds to cholesterol at the raft periphery and uses its cytoplasmic tail to recruit M1, already attached to vRNPs, at the assembly site [13], before inducing particle budding and release [2]. Thus, M1 localization at the budding site could be the result of an electrostatic and hydrophobic interaction with plasma membrane lipids [14] or/and of interactions with the cytoplasmic tail of HA, NA [15] or M2 [13] [16]. As M2 cytoplasmic tail includes negatively charged amino-acids and M1 incorporation in virions is decreased upon M2 mutation, Chen and colleagues hypothesized the presence of an electrostatic interaction between M2 and M1 [13]. The M1 residues that specifically interact with the plasma membrane have not yet been identified, but they should be positively charged [17]. However, the involvement of M1 basic residues in this process is debated [18]. M1 has two main domains: an N-terminal domain composed of the first 164 amino acids and a C-terminal domain composed of amino acids 165 to 252. The C-terminal domain has been involved in M1-vRNP interaction *in vitro* [17] and is essential for M1 multimerization and incorporation in viral particles [19]. In the A(H1N1)pdm09 strain, M1 N-terminal domain includes two basic motifs: an arginine triplet (R76/77/78) on helix 5 and the Nuclear Localization Signal (NLS; 101-KKLKR-105) on helix 6 (Fig 1). The NLS, first described by Ye *et al*. [20], is needed for M1 translocation in the nucleus during the late steps of viral replication. Once in the nucleus, M1 interacts with the vRNPs (via NP) and NEP. NEP recruits the cellular factor CRM1/exportin1 for nuclear export of the vRNP complexes in the cytoplasm [21]. The interaction of M1 with vRNPs and NEP was proposed to "hide" M1 NLS and consequently to prevent its return into the nucleus. The positively charged NLS could also participate in M1/membrane interaction [17], but this remains controversial [18]. The arginine triplet in position R76/77/78 was first described by Das *et al*. in 2012 in another influenza A strain and is highly conserved among influenza A and B viruses [22]. Mutation of one or two of the three arginine residues reduces virus production due to a budding defect, apparently caused by accumulation of M1-containing vesicles below the cell membrane [22]. To unravel the role of these basic residues in the late steps of the virus live cycle, we decided to further investigate the role of these two M1 basic domains in influenza A(H1N1)pdm09 virus assembly and release.

## 2-Materials and Methods

### Plasmids and site-directed mutagenesis

PolI/PolII pHW2000 plasmids containing the sequences of the different influenza A/Lyon/969/2009(H1N1) segments were kindly provided by M. Ottmann and B. Lina (Lyon University). The Genbank accession numbers of the different segments are: KC800977 (segment 1/PB2), KC800978 (segment/PB1), KC800979 (segment 3/PA), JF429402 (segment 4/HA), KC800980 (segment 5/NP), JF429403 (segment 6/NA), KC800981 (segment 7/M) and KC800982 (segment 8/NS). Eukaryotic expression vectors were obtained by subcloning the coding sequences of M1 and M2 from the pHW2000 M plasmid in pcDNA3-hygro(-) plasmid. Substitution mutations were introduced in M1 NLS (101-K/RKLKR-105) and arginine triplet (76-RRR-78) by site-directed mutagenesis using the QuickChange mutagenesis kit (Agilent), according to the manufacturer's protocol (Fig 1). Alanine residues replaced the basic arginine residues 76, 77 and 78 of the triplet motif (R77A, R77/78A and R76/77/78A) and the basic lysine residues 101 and 102 of the NLS (K101/102A). Moreover, the three amino acids at positions 74 to 76 of the cytoplasmic tail (CT) of M2 from the influenza A(H1N1)pdm09 strain were also replaced by alanine residues (E74EY76->AAA, called M2-mut2), as reported elsewhere [13]. All plasmids were amplified in *E*. *coli* and mutations were confirmed by DNA sequencing (MWG Eurofins). To detect M2 in cells, a fluorescent fusion protein was created by fusing GFP or mCherry with the C-terminus of M2. For fluorescence microscopy, RAB11-mRFP or PH-PLCd-GFP were used to label recycling endosomes or the cell plasma membrane, respectively.

### *In vitro* M1 matrix protein purification

The prokaryotic expression vector pET16b encoding M1 N-terminal domain (1–164) was kindly provided by W. Weissenhorn (Grenoble). Site-directed mutagenesis was performed, as described above, to obtain the M1 mutant R76/77/78A. As the available anti-M1 antibody does not recognize the N-terminal domain, a poly-His Tag was added to the C-terminal sequence of both wild type (M1 WT) and mutated M1 N-terminal domain (M1 R76/77/78A) for detection by immunoblotting. BL21 cells were transformed with 100ng of plasmid and grown in Luria Broth Medium supplemented with ampicillin. After cultures reached a OD = 0.45–0.55 at 600 nm, M1 expression was induced by adding 0.5mM isopropylthiogalactopyranosid (IPTG) and 4h post-induction cells were harvested and resuspended in 10mM NaCl/Tris buffer, pH 7.6 (M1 WT) or pH 6 (M1 R76/77/78A). After sonication, cell suspensions were centrifuged at 20000g at 4°C for 15min and supernatants containing the proteins were purified by fast protein liquid chromatography (FPLC) with a cationic exchange column (HiTrap, Sephadex) by increasing NaCl concentration. Proteins were then directly used for the experiments or stored at -20°C.

## *In vitro* co-sedimentation assays with large unilamellar vesicles

Binding of M1 WT and M1 R76/77/78A to negatively charged lipids was determined by co-sedimentation assays with large unilamellar vesicles (LUVs). LUVs were made of a mixture of Egg-Phosphatidylcholine (EPC), brain Phosphatidylserine (PS), Phosphatidylinositol-4,5-biphosphate (PI(4,5)P2) and cholesterol at two different molar ratios (20:50:0:30 or 26:42:2:30 mol:mol:mol:mol). All lipids were provided by Avanti Polar Lipids. Lipid mixtures were solubilized in chloroform and dried by evaporation. Lipids were then resuspended overnight in KCl/Hepes buffer (150mM KCl, 0.5mM EDTA, 20mM Hepes, pH 7.4) and extruded to obtain LUVs of 200nm in diameter, as measured by dynamic light scattering (Zetasizer Nano Series ZS, Malvern Instruments). A constant amount (4 μg) of recombinant M1 WT or R76/77/78A was incubated with LUVs (1:150 M1: PS± PI(4,5)P2) in a final volume of 100μL at room temperature for 10min. Samples were then centrifuged at 42000rpm in a Beckman TLA 110 rotor at 4°C for 30min. Each sample was then divided in supernatant (S = 90 μl), containing unbound M1, and pellet (P = 10 μl), containing LUV-bound M1. P was diluted in 80 μL of KCl/Hepes buffer to maintain the equivalence between the S and P volumes. Then, 20 μL of S and P were analyzed by SDS-PAGE and M1 was detected by staining with Coomassie Blue or by western blotting using an anti-His tag antibody (Thermo Scientific). The M1 intensities (I_s_, I_p_) were quantified using the Image J software (http://imagej.nih.gov/ij/). The percentage of LUV-bound M1 was calculated as: % M1 LUV-bound = 100*I_P_/(I_P_+I_S_).

### Cell culture and transfection

The human embryonic kidney (HEK) 293T cell line used in this study was maintained in Dulbecco's modified Eagle medium (DMEM) supplemented with 10% fetal calf serum (FCS), complemented with sodium pyruvate and antibiotics (penicillin-streptomycin), at 37°C with 5% CO_2_. Madin-Darby canine kidney (MDCK) cells were maintained in DMEM supplemented with 10% FCS (Sigma) and 1% penicillin-streptomycin (Thermo) at 37°C and 5% CO_2_ atmosphere.

293T cells were transfected using the calcium phosphate technique. Based on the work by Chen *et al*. [23] and adapted to our conditions, plasmids were transfected as follow (2.10^6^ cells/transfection): pcDNA-M1, 8 μg; pcDNA-M2, 2 μg; pHW2000-M, 2 μg; pHW2000-NS (which codes for NS1 and NEP), 2 μg; pHW2000-NP, 8 μg; pHW2000-HA, 2 μg; and pHW2000-NA, 2 μg. The amount of transfected plasmid was normalized by adding pcDNA3 empty plasmid. The cell medium was replaced 24h post-transfection and experiments were performed 48h post-transfection. Cell transfection efficiency (M1 or M2) was assessed by fluorescence microscopy with an anti-M1 secondary tagged fluorescent antibody or based on mCherry fluorescence (M2) and was calculated as the number of fluorescent (transfected) cells relative to the total number of cells (evaluated by counting the DAPI-colored nuclei) x100. For each experiments, transfection efficiency was 70% for M1 (for 50 <n< 80 cells) or M2.

### Virus rescue

An 8-plasmid DNA transfection system was used as described previously [24]. Briefly, the eight PolI/PolII reverse genetic plasmids encoding the eight influenza virus segments (0.5 μg each) were transfected in HEK 293T cells plated in 6-well plates using Lipofectamine3000 (Thermo Scientific). After 24h, transfected cells were removed from the wells and co-cultured with MDCK cells in 25-mL flasks. For the first 8 hours, cells were co-cultured in 10% serum and then medium was replaced by serum-free medium containing 0.5μg/mL TPCK-treated trypsin (Sigma Aldrich). Supernatants at day 5 post-transfection were used for virus amplification in MDCK cells in a T75 flask. Viral titers were then determined by plaque assay in MDCK cells.

### Antibodies

Immunoblots were performed using the following antibodies: rabbit anti-GFP (Life Technologies), rabbit anti-M1 (GeneTex), rabbit anti-NS1 and anti-NEP (Thermo Scientific), mouse anti-tubulin and mouse anti-LAMP2 (Life Technologies), goat anti-S6 (Santa Cruz Biotechnologies), and anti-mouse, anti-rabbit and anti-goat secondary antibodies coupled to horseradish peroxidase (Dako). For HA detection, an influenza A(H1N1)pdm09 immunized human serum, provided by Y. Mekki (Service de Virologie du Centre de Biologie et de Pathologie Nord, HCL, Lyon, France). For vesicular cell marker detection by immuno-fluorescence, goat anti-EEA1(N-19), mouse anti-CD63 (MX49) and mouse anti-LAMP2 (H4B4) antibodies (Santa Cruz Biotechnologies) and a mouse anti-LC3 (a gift from L. Espert, Montpellier) and fluorescent Alexa® 488 or 555-conjugated donkey, mouse, rabbit or goat secondary antibodies (Molecular Probes, Invitrogen) were used.

### Western blot analysis

For western blot analysis, 50 μg of each protein samples, 20 μL of membrane flotation assay fractions or 20 μL of VLP preparations were mixed with SDS loading buffer, separated on 10% SDS-PAGE gels and transferred to polyvinylidene difluoride membranes. Immunoblotting was performed using the relevant antibodies. Horseradish Peroxidase coupled secondary antibodies were detected with the SuperSignal West Pico or Femto substrate (Thermoscientific). The resulting signals were imaged with a G:Box (Syngene).

### Virus-like particle (VLP) purification

Culture supernatants containing VLPs were harvested 48h post-transfection, filtered (0.45μm pores) and centrifuged on a cushion of 30% sucrose in TNE buffer (10mM Tris-HCl pH 7.4, 100mM NaCl, 1mM EDTA) in a Beckman SW41Ti rotor at 200000g and 4°C for 2h. Pellets were resuspended in TNE buffer at 4°C overnight and VLP presence was checked by western blotting. To estimate VLP release, M1 signal in the blots was quantified using the ImageJ software. The percentage of M1-containing VLPs released in the supernatant was calculated as follow: % of M1-containing VLPs = M1_released_/(M1_released_+M1_intracellular_).

### Membrane flotation assay

For each condition, 6x10^6^ cells were transfected and viral supernatants harvested 48h post- transfection, as described above. Cells were washed with ice-cold PBS and resuspended in Tris-HCl containing 4mM EDTA and 1X Complete protease inhibitor cocktail (Roche). Every step was then performed at 4°C. Cell suspensions were lysed using a Dounce homogenizer, then centrifuged at 600g for 3min to obtain Post-Nuclear Supernatants (PNS). A cushion of 820μL of 75% (wt/vol) sucrose in TNE buffer (25mM Tris-HCl, 4mM EDTA, 150mM NaCl) was loaded at the bottom of an ultracentrifuge tube and mixed with 180 μL of PNS adjusted to 150mM NaCl. Two milliliters and 300 μL of 50% (wt/ml) sucrose cushion followed by 0.9 mL of 10% (wt/ml) sucrose cushion were then layered to obtain the gradient that was then centrifuged in a Beckmann SW60Ti rotor at 35 000rpm, 4°C, overnight. Eight 500μL fractions were collected from the top to the bottom of the centrifuge tube and analyzed by western blotting.

### Subcellular fractionation

The first steps of subcellular fractionation correspond to the steps described above, except that when PNS were obtained by centrifugation at 600g for 3min, pellets containing the nuclear fraction were kept, washed once and resuspended in 100 μl of TNE buffer. Then, 100 μL of PNS were centrifuged at 10000g for 10min to separate the cytosol (supernatant) from the cell membranes fraction (pellet). Different fractions were obtained (nuclei, PNS, cytosol, cell membranes). Protein concentration was measured using the Bradford method (Coomassie Blue Protein Assay Reagent, Thermo Scientific) and samples were analyzed by immunoblotting. Signal intensity was quantified using the ImageJ software and normalized to the sample volume and/or to the intracellular beta tubulin signal, as appropriate.

### Immunofluorescence and confocal microscopy

Cells were grown on polylysine-coated coverslips and transfected 24h later, as described above. Forty- eight hours post-transfection, cells were fixed in 3% paraformaldehyde/PBS for 15min and washed with 50mM NH_4_Cl buffer to remove the fixative and to quench free aldehydes. Then, cells were permeabilized with 0.2% Triton X-100 for 5min, incubated in blocking solution (1% BSA) for 15min and then with the anti-M1 rabbit antibody (GeneTex) followed by the secondary antibody, as described above. Images were acquired using a LSM780 confocal microscope (Zeiss) and an Apochromat 63x oil objective, supplied with the Zen Software. When performed, z-stacks were piles of 1 μm depth images.

### Electron microscopy (EM)

Cells were fixed in 4% paraformaldehyde and 1% glutaraldehyde in 0.1M phosphate buffer (pH 7.2) for 48h, washed with PBS, post-fixed in 1% osmium tetroxide for 1h and dehydrated in a graded series of ethanol solutions. Cell pellets were embedded in EPON™ resin (Sigma) that was allowed to polymerize at 60°C for 48h. Ultrathin sections were cut, stained with 5% uranyl acetate and 5% lead citrate and deposited on colloidon-coated EM grids for examination using a JEOL 1230 transmission electron microscope.

## 3-Results

### The M1 arginine triplet is essential for M1 N-terminal domain interaction with negatively charged model membranes *in vitro*

To analyze the role of the M1 arginine triplet R76/77/78 (Fig 1) in M1 interaction with membranes, we first checked *in vitro* whether mutations in this motif could prevent M1 attachment to model membranes. To this aim, the N-terminal domain of the wild type influenza A(H1N1)pdm09 M1 matrix protein (1–164; M1 WT) and the corresponding mutated version (M1 R76/77/78A) were expressed in bacteria, purified and their interaction with LUVs assessed by co-sedimentation assays.

**Fig 2:**
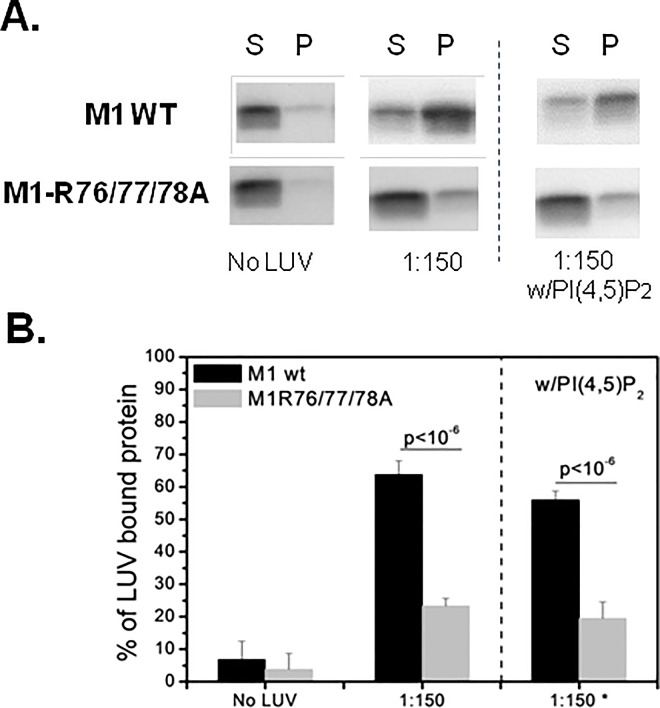
Binding of the recombinant N-terminal domain of M1 (wild type and Arg triplet mutant) assessed by LUV co-sedimentation assays. “M1” corresponds to the N-terminal M1 domain (first 1–164 amino-acids). After ultracentrifugation, supernatants (S, containing free M1) and pellets (P, containing LUV-bound M1) were loaded on SDS-PAGE gels and M1 presence in S and P was quantified using the ImageJ software to obtain the percentage of M1 bound to LUVs by the following formula: % of M1 LUV-bound = 100*I_P_/(I_P_+I_S_). The results are representative of five independent experiments. The differences between M1 WT and M1-R76/77/78A were estimated using the Student’s t test (p-value indicated in the figure). (A) Immunoblotting with anti-M1 antibodies showing M1 WT or mutant (R76/77/78A) expression in the S and P fractions. 1:150, LUVs made of PC:PS:cholesterol (20:50:30) with a constant M1 to PS ratio (1:150); w/PI(4,5)P_2_, LUVs containing also 2% of PI(4,5)P_2_. (B) Percentage of M1 WT or mutant (R76/77/78A) bound to LUVs containing cholesterol, PS and PC with or without PI(4,5)P_2_ (as in A) at the M1:PS molar ratio of 1:150. (*) LUVs with PI(4,5)P_2_.

Using LUV-protein binding assays, Ruigrok *et al*. [14] showed that the binding of M1 N-terminal domain to LUVs mainly occurs when PS and cholesterol represent 50% of all membrane lipids. Moreover, M1 does not bind to LUVs when PS is reduced to 25%. On the basis of this finding and the work by Baudin *et al*. [17] where PS represented 50% of the total lipids in LUV composition, we used LUVs made of PC:PS:cholesterol/20:50:30. We maintained the PS to M1 ratio (150:1, i.e., a LUV excess) and the LUV lipid molar ratio constant. Compared with M1 WT (64±4%) (black bars), M1 R76/77/78A binding to LUVs (gray bars) was reduced by three-fold (23±2%) (p<10^−6^), but not completely abolished (Fig 2A and 2B). We supposed that this residual binding could be attributed to the basic NLS domain of M1 (particularly the basic charged amino acids, position 101–105) (Fig 1). Unfortunately, we could not produce and purify the double mutant protein containing both the M1 R76/77/78A and NLS K101/102A mutations to confirm this point, due to insufficient protein expression level.

Although PS is a component of the plasma membrane, it is also detected in the Golgi and in the endoplasmic reticulum (reviewed in [25]). Conversely, the phospholipid PI(4,5)P2 is exclusively found in the inner leaflet of the plasma membrane, the site of influenza A virus assembly. To test the potential role of PI(4,5)P2 in M1 binding to membranes, we introduced 2% of PI(4,5)P_2_ in the LUV lipid composition and concomitantly decreased the PS molar ratio to keep constant the surface charge per LUV. The presence of PI(4,5)P2 did not change M1 WT or M1 R76/77/78A binding to LUVs (Fig 2A, w/PIP2 lane; and in Fig 2B, w/PI(4,5)P_2_ panel). Altogether, these results indicate that M1 interaction with LUVs requires the R76/77/78 motif and is not improved by PI(4, 5)P2.

### The viral proteins M2+NS1/NEP and the M1 arginine triplet are essential for M1 localization in cell membrane fraction

**Fig 3:**
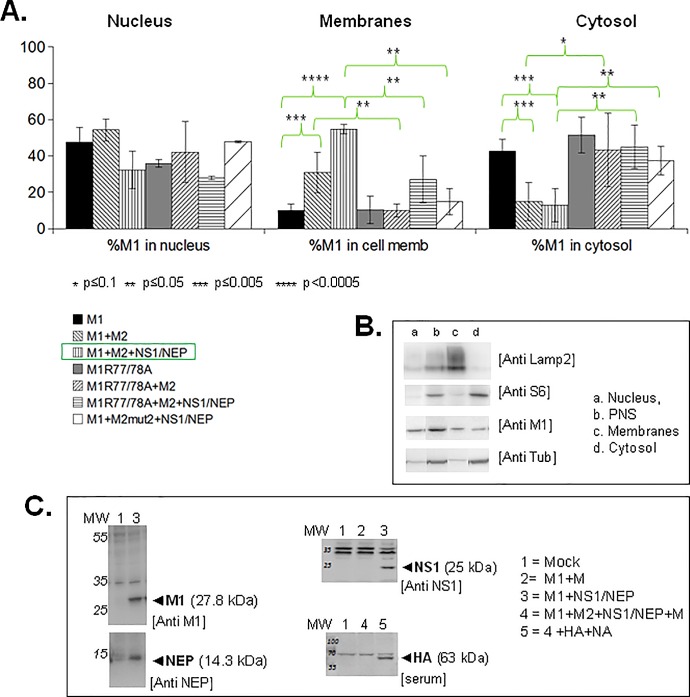
Minimal viral partners and M1 basic residues essential for influenza A(H1N1)pdm09 M1 membrane localization. HEK 293T cells were transfected with empty vector (mock) or with the pcDNA-M1 (M1 WT or mutant), pcDNA-M2 (M2), pHW2000-NS (NS1/NEP) or pHW2000-M (M) plasmids, as indicated. Cell fractionation experiments were performed 48h post-transfection. (A) Percentage of M1 detected in the nuclear, cell membrane or cytosolic fraction in each condition. M2-mut2 was used as control for low M1 membrane binding. The histograms show the result of at least three independent experiments (mean± standard deviation represented in the error bars). Differences between conditions were assessed using the Student’s t-test. (B) Cell fractionation controls. Fractions of cells co-expressing M1+M2+NS1/NEP were immunoblotted with antibodies against a membrane marker (LAMP2) and a cytosolic marker (the ribosomal S6 protein). Tubulin was used as loading control. PNS, Post-Nuclear Supernatant. (C) Expression of the indicated influenza A(H1N1)pdm09 viral proteins was checked after transfection in HEK 293T cells of the relevant plasmids by western blotting with anti-M1 (H1N1), anti-NEP and anti-NS1 antibodies. HA was detected with a serum obtained using an influenza A(H1N1)pdm09 strain isolated from a vaccinated patient.

**Fig 4:**
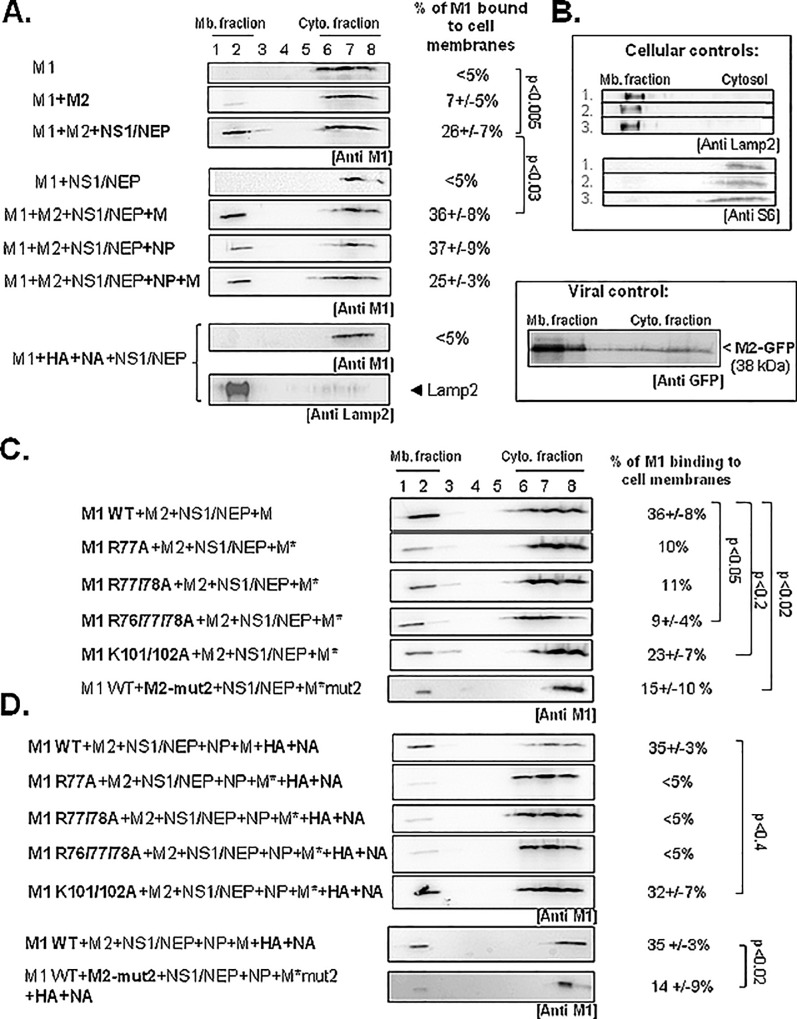
Minimal viral partners and M1 basic residues essential for A(H1N1)pdm09 M1 membrane attachment using cell membrane flotation assay. HEK 293T cells were transfected with empty vector (mock) or with pcDNA-M1 (WT or mutants), pcDNA-M2, pHW2000-NS, pHW2000-M, pHW2000-NP, -HA, or–NA, as indicated. M2-mut2, an M2 CT mutant was used as control for low M1 membrane binding. M*, M harboring the relevant mutations in M1 or M2 coding sequences. (A) Membrane flotation assays were performed as described in Methods. (B) LAMP-2 and S6 were used as, respectively, membrane and cytosolic fraction markers. Expression of the fusion protein M2-GFP (localized in the membrane fraction) was detected with an anti-GFP antibody. (C) Analysis by membrane flotation assays of the effect of M1 basic motif mutations on M1 membrane attachment. (D) Membrane flotation assays performed in the presence also of NP, HA and NA. The percentages of membrane-bound M1 are the mean ± standard deviation of three independent experiments (except for M1R77A and R77/78). The p values indicate significant differences relative to the minimal system. Mb, membrane; Cyto, cytosolic.

To explore the role of the R76/77/78 basic motif in M1 interaction with cell membranes, we first determined whether mutations in this motif could prevent M1 localization in cell membrane fraction. Forty-eight hours after transfection in HEK 293T cells of the pcDNA-M1 and pcDNA-M2 (C-terminally tagged with GFP) plasmids and of PolI/PolII plasmids (reverse genetic approach) to express the other influenza A(H1N1)pdm09 proteins (see Material and Methods), we analyzed M1 cell localization (nucleus, cytosol and cell membrane) in the presence or not of the other viral proteins, by subcellular fractionation followed by western blots (Fig 3A and 3B). Immuno-fluorescence labeling of fixed transfected cells with an anti-M1 antibody confirmed the transfection efficiency (about 70% for n = 76 cells). Analysis of the expression of the A(H1N1)pdm09 influenza proteins by immunoblotting showed that M1, HA, NEP and NS1 were expressed in HEK 293T cell lysates (Fig 3C). We checked M2 expression at the cell membrane by membrane flotation assay and an anti-GFP antibody (Fig 4B, lower panel).

M1 quantification in the different subcellular fractions by ImageJ after M1 detection by western blotting (as an example, see Fig 3A and 3B), as described in Material and Methods, showed that M1 alone was mainly located in the nucleus (47±8%) and in the cytosol (42±7%). When co-expressed with M2 (M1+M2), the amount of M1 in the cell membrane fraction increased to 31±11%. In the presence of M2+NS1/NEP, up to 55±3% of M1 was in the cell membrane fraction, while the percentage in the cytosolic fraction decreased to 13±9%. Since the nuclear M1 (~40%) is barely affected by the expression of M2+NS1/NEP, our data suggest that one important part of the cytosolic M1 is re-localized to the membrane fraction. We used cell fraction markers (LAMP2 for labelling the membrane fraction; S6 for the cytosol fraction) (Fig 2B) to confirm M1 localization in the membrane fraction upon addition of M2 and NS1/NEP. The M1 nuclear fraction did not change much in the different conditions confirming the capacity of M1 to shuttle between the cytoplasm and the nucleus. Our results show that M1 associated to cell membranes is promoted by the expression of NS1/NEP (segment 8) and an overexpression of M2. As influenza A virus NS1 can stimulate M1 translation, its presence could promote M1 cell concentration and consequently its detection at the cell membrane. Moreover, Chen *et al*. [13] and Wang *et al*. [26] reported a possible M1-M2 interaction. Therefore, M2 overexpression could favor M1 localization at the plasma membrane where M2 is mainly found.

To examine the involvement of the M1 arginine triplet in M1 cell membrane localization, we overexpressed the M1 R77/78A double mutant alone or with M2 and NS1/NEP before subcellular fractionation (Fig 3A). The cell localization of M1-R77/78A alone was comparable to that of M1 WT alone. M1 R77/78A co-expressed with M2 was mainly in the nuclear (42±16%) and cytosolic fractions (43±20%). The percentage of M1-R77/78A in the cell membrane fraction did not change upon co-expression with M2 (10±3% with and without M2), differently from M1 WT (31±11%). Moreover, when NS1 and NEP were co-expressed with M1-R77/78A and M2, only 27±13% of M1 R77/78A was in the cell membrane fraction (compared with up to 55±3% for M1 WT). The percentage of M1 R77/78A in the cytosolic fraction did not vary in the presence of M2 and NS1/NEP (45±11%), as observed for M1 WT (Fig 3A). As a control for the low binding of M1 to cell membranes, we used a previously described A(H1N1)pdm09 M2 CT mutant (M2-mut2; EEY74-76AAA mutant) [13]. Upon co-expression with M2-mut2, the amount of M1 in the cell membrane fraction was reduced by three-fold compared with M2 WT (15 ±7% and 55±2%, respectively) (Fig 3A). In the presence of M2-mut2, M1 loose its membrane binding properties, confirming the role of M2 in the recruitment of M1 at the plasma membrane. Altogether these data indicates that the M1 arginine triplet is not a key player in M1 nucleus-cytosol trafficking control, but rather in M1 localization at the cell membrane through interaction with and/or in the presence of M2.

### The M1 arginine triplet is essential for M1 attachment to the cell membranes

To determine whether M1 N-terminal basic motifs have a direct role in M1 interaction with the cell membrane, we performed membrane flotation assays (Fig 4A and 4B). Using our minimal experimental system with the addition of segment 7 (M) for optimal M1 membrane detection (i.e., M1+M2+NS1/NEP+M), we investigated the effect of mutations in the M1 arginine triplet (M1 R76/77/78A) or in the NLS (K101/102A) on M1 attachment to cell membranes by quantification following western blot analysis of the different fractions obtained by subcellular fractionation, as described in Materials and Methods. We used S6 (cytosolic ribosomal protein) and LAMP2 (a lysosomal membrane protein) as controls for the cytosolic and membrane fractions, respectively (Fig 4B). When only M1 was expressed, we detected 10±3% of M1 in the cell membrane fraction by subcellular fractionation (Fig 3A), but none by membrane flotation assays (3±3%, i.e., <5%, Fig 4A, panel M1). M1 membrane attachment increased by 2-fold upon co-expression with M2 (7±5%, panel M1+M2, Fig 4A) and by 9-fold upon co-expression with M2, NS1 and NEP (up to 26±7%, Fig 4A). When M2 was not co-expressed, M1 remained cytosolic even in the presence of NS1 and NEP, (Fig 4A, panel M1+NS1/NEP). In addition, co-expression of HA/NA instead of M2 did not restore M1 membrane localization (Fig 4A, panel M1+HA+NA+NS1/NEP). In these different conditions, we confirmed HA expression in the cytosol and membrane fractions (PNS) by western blotting (S1 Fig). Thus, in HEK 293T cells, co-expression of at least M2+NS1/NEP is essential for M1 localization at the cell membranes. Moreover, the additional expression of M (pHW2000-M plasmid) increased to 36±8% the amount of M1 bound to cell membranes (Fig 4A, panel M1+M2+NS1/NEP+M), probably due to the increase of intracellular M1. Co-expression of NP or NP+M (in addition to M2 and NS1/NEP) did not further change the percentage of M1 bound to cell membranes.

On the other hand, membrane flotation assays following co-expression of M1 arginine triplet mutants with M2, NS1/NEP and M* (pHW2000-M* in which the M1 sequence was also mutated) showed that the amount of M1 mutant bound to cell membrane was reduced by at least 3-fold (~10% for M1 R77A and for M1 R77/78A, and 9±4% for M1 R76/77/78A) compared with M1 WT (36±8%) (Fig 4C). To check whether the other conserved M1 basic motif had a role in M1 membrane attachment, we used the M1 NLS K101/102A, mutant. Compared with M1 WT co-expressed with M2+NS1/NEP+M (36±8%), M1 NLS K101/102A cell membrane attachment was reduced (23±7%), but to a lesser extent than with the arginine triplet mutants (Fig 4C). As a control for low binding of M1 to cell membrane, we used M2-mut2 (Fig 4C and 4D). M1 cell membrane attachment was strongly reduced upon expression of M2-mut2 compared with wild type M2 (15±10% and 36±8%, respectively) (Fig 4C), similar to what observed with the M1 arginine triplet mutants.

It has been reported that expression of the envelope proteins HA and NA can influence M1 membrane localization (6). In our experimental condition, co-transfection of HA and NA with M1+M2+NS1/NEP+NP+M slightly increased M1 membrane attachment compared with M1+M2+NS1/NEP+NP+M alone (35±3% and 25±3%, respectively; p <0.01) (Fig 4D). This suggests that in our assay, HA and NA can improve M1 cell membrane attachment, but not when M1 carries mutations in the arginine triplet (<5%) (Fig 4D). M2-mut2 also reduced M1 cell membrane attachment in the presence of HA and NA (14±9%). Conversely, attachment of M1 K101/102A to the cell membrane was similar to M1 WT (32±7% and 35±3%, respectively) (Fig 4D). Western blot analysis showed that HA expression was comparable when co-expressed with M1 WT or M1 K101/102A and was a little bit higher with M1 R76/77/78, or in the absence of M2 (S1 Fig). Thus, in our influenza A(H1N1)pdm09 virus minimal experimental system, M1 cell membrane attachment requires the R76/77/78 motif, but not the NLS K101/102 motif, as suggested in [18]. The EEY motif of M2 CT also is essential for M1 cell membrane localization, as described previously for the influenza A/Udorn/72 strain [13]. Addition of the viral proteins M, NP and HA/NA is not essential for M1 binding to cell membranes, and HA cannot replace M2 in that function.

### M1 arginine triplet mutant accumulates in intracellular clusters

**Fig 5:**
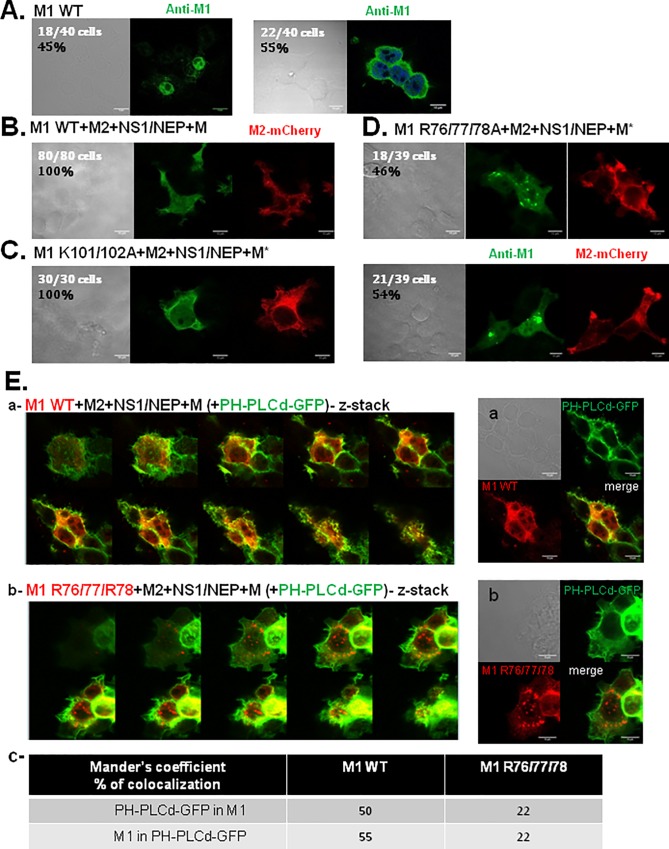
Cellular localization of M1 and its basic mutants using immunofluorescence confocal microscopy. Immunofluorescence confocal microscopy imaging of HEK 293T cells transfected with pcDNA-M1 (WT or R76/77/78A), pcDNA-M2-mCherry and pHW2000-NS1/NEP, as indicated (A, B, C and D). M1 was detected using a primary anti-M1 antibody and a secondary antibody coupled to Alexa488 (in green), M2-mCherry is shown in red. Transmission images are in grey. Scale bars, 10 *μ*m. (E) Analysis of M1 WT and M1 R76/77/78A localization at the plasma membrane using the PH-PLCdelta-GFP membrane markers. (a) and (b) Immunofluorescence confocal microscopy z-stack images of HEK 293T cells transfected with pcDNA-M1 (WT in (a) and R76/77/78A in (b)), pcDNA-M2, pHW2000-NS1/NEP and M + PH-PLCd-GFP. M1 was detected using a primary anti-M1 antibody coupled to an Alex555 secondary antibody (in red). GFP is in green. Transmission is in grey. Scale bar, 10*μ*m. (c) Co-localization quantification of the M1 signal with PH-PLCd-GFP (Mander’s overlap coefficients).

We then analyzed M1 cellular localization by immunofluorescence coupled to confocal microscopy in HEK 293T cells that express M1 WT, M1 K101/102A or M1 R76/77/78A and M2-mCherry, NS1/NEP and M (or M* in the case of M1 mutants) (Fig 5). In cells that expressed only M1 WT (Fig 5A), M1 was localized in the nucleus (45%) and/or in the cytosol (55%). In cells that co-expressed M2-mCherry, NS1/NEP and M, M1 WT was localized in the nucleus, cytosol and at the plasma membrane (Fig 5B). Expression of the M1 K101/102A mutant did not change the overall localization of M1. Indeed, like M1 WT, M1 K101/102A was in the cytosol and partly at the plasma membrane with a membrane labeling similar to that of M2-mCherry (Fig 5C). The co-localization of M1 WT with the plasma membrane was observed with the PH-PLCd-GFP marker (Mander’s overlap coefficient = 50 to 55%) (Fig 5E, a, c). Conversely, the M1 R76/77/78A mutant was mainly in intracellular aggregates (Fig 5D) that did not co-localize, or very little, with the plasma membrane (Fig 5E, b, c; Mander’s overlap coefficient = 22%) nor with different vesicular compartments (S2A Fig, c-e and S2B Fig), such as late endosomes, lysosomes or autophagosomes (Mander’s overlap coefficients for all vesicular compartments were lower than 10%, indicating no co-localization). A ~20% co-localization of M1 R76/77/78 was observed with early or recycling endosomes (S2A Fig, b and a, respectively, and S2B Fig), suggesting a low probability for M1 mutant to locate in these compartments. These results strongly suggest that mutation in the M1 arginine triplet prevents M1 cell membrane localization, in agreement with the results obtained with the membrane flotation assays (Fig 4C).

### The M1 arginine triplet is essential for M1 incorporation in Virus-Like Particles and for influenza A(H1N1)pdm09 virus infectivity

**Fig 6:**
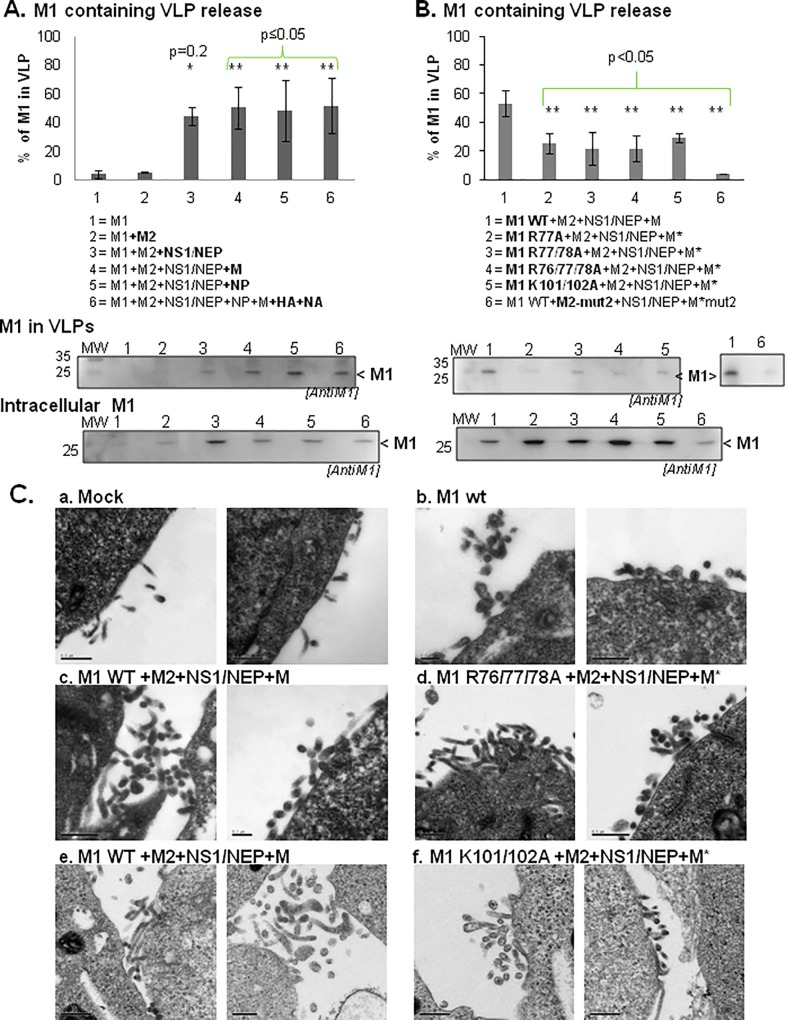
VLP production in the presence of different influenza A(H1N1)pdm09 viral proteins and M1 mutants. Cell supernatants were centrifuged on a sucrose cushion and VLPs were resuspended in TNE buffer. M1 (intracellular and in VLPs) was detected by western blotting using an anti-M1 antibody. M1 release was calculated using the following formula: % of M1 released = M1 in VLPs / (M1 in VLPs + intracellular M1) and the results are the mean ± standard deviation (error bars) of three independent experiments. Significant differences between condition 2 (M1+M2) in 6A and condition 1 (M1 WT+M2+NS1/NEP+M) in 6B and the other conditions were calculated by using the Student’s t test: *, p = 0.2, ** p ≤0.05. (A) Minimal partners required for the production of M1-containing VLPs. (B) M1-containing VLP production upon expression of M1 WT or mutants. The M2 CT mutant M2-mut2 was used as control. (C) Electron microscopy analysis of influenza A(H1N1)pdm09 VLP production in HEK 293T cells. Cells were transfected with pcDNA empty vector (Mock) or pCDNA3-M1 (WT or mutants), +/- pcDNA-M2, pHW2000-NS and pHW2000-M (M* bearing the indicated M1 mutations), as indicated: Mock (a), M1 WT alone (b), M1+M2+NS1/NEP+M (c and e), M1R76/77/78A+M2+NS1/NEP+M* (d) and M1 K101/102A+M2+NS1/NEP+M* (f). Scale bars: 0.5 *μ*m, except for the left panel in b and the right panel in c where the scale bars represent 0.2 *μ*m. These experiments were done three times independently using new batches of transfected cells.

To determine whether in our experimental system, M1 presence at the cell plasma membrane was correlated with VLP production, we measured the release of M1-containing VLPs by immunoblotting (Fig 6A and 6B) and VLP budding at the cell plasma membrane by EM (Fig 6C) following transfection of HEK 293T cells with the indicated plasmids. Quantification of the amount of pelleted VLPs that contained M1 (see Materials and Methods) showed that when M1 was expressed alone or with M2, M1-contained in pelleted VLPs represented only 3±3% and 4±3% of all M1, respectively (Fig 6A; lanes 1 & 2, in the histogram and anti-M1 immunoblots). This amount increased to 38±12% in the presence of M2+NS1/NEP (Fig 6A, lane 3). Co-expression also of M (lane 4), NP (lane 5) and HA+NA (lane 6) did not further increase the amount of M1-containing VLPs in the pellet (35±14%, 37±8% and 43±13% respectively) (Fig 6A). Thus, the minimal system to produce M1-containing VLPs is M1+M2+NS1/NEP±M, in agreement with the results of the membrane flotation assays (Fig 4) and M1 cell membrane localization (Figs 3 and 5). This was also confirmed by VLP production visualization by EM (Fig 6C). We could observe budding events only in HEK 293T cells expressing M1+M2+NS1/NEP+M (Fig 6C, c and e), but not in mock-transfected cells (Fig 6C, a) and in cells expressing M1 alone (Fig 6C, b).

Co-expression of M1 arginine triplet mutants with M2, NS1/NEP and M (M* carried the corresponding mutation in the M1 or M2 coding sequence) strongly reduced M1-containing VLPs (M1 R77A: 25±75%; M1 R77/78A: 21±11%; M1 R76/77/78A: 21±9%) compared with M1 WT (54±9%) (Fig 6B, lanes 1 to 4). We obtained similar results also with M1 K101/102A (NLS mutant) (27±10%; 2-fold less than with M1 WT). As M1 WT membrane binding was reduced when co-expressed with M2-mut2 (Fig 4C and 4D), we also checked the effect of M2-mut2 on VLP production (Fig 6B, lane 6; M1+M2-mut2+NS1/NEP+M-mut2). M2-mut2 co-expression decreased M1 incorporation in VLPs by 10-fold (4±0.01%) compared with M2 WT (Fig 6B, compare lane 6 and 1). Our results are in agreement with the finding that M2 is involved in M1 incorporation in VLPs [13] [26]. Altogether, these results indicate that both M1 basic motifs are involved in the release of M1-containing VLPs or in M1 incorporation in VLPs.

EM analysis showed that VLP formation and budding at the cell surface were comparable in HEK 293T cells that expressed M1 R76/77/78A+M2+NS1/NEP+M* (Fig 6C-d), M1 K101/102A+M2+NS1/NEP+M* (Fig 6C-f) or M1 WT+M2+NS1/NEP+M (Fig 6C-c or e). Similarly, the mean VLP diameter (measured in EM images) was 130±30nm, 120±20nm and 130±30nm for M1 WT-, M1 R76/77/78A- and M1 K101/102A-containing VLPs, respectively, (from three independent experiments; 10 to 15 VLPs analyzed). This suggests that the M1 R76/77/78A mutation affects M1 incorporation in VLPs rather than VLP formation, in agreement with the results by Das *et al*. [22] using the influenza A/WSN/33(H1N1) strain. However, we cannot totally exclude that this mutation might also affect particle release in the cell culture supernatant.

**Fig 7:**
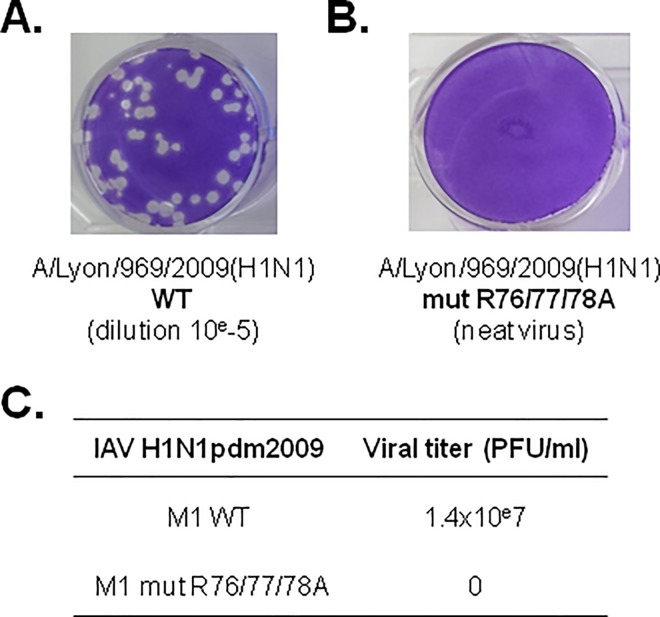
Infectivity of influenza A(H1N1)pdm09 strains carrying M1 WT or M1 R76/77/78A. A plasmid reverse genetic approach was used to rescue pH1N1 (A/pdm09) carrying M1 WT or M1 R76/77/78A. (A) The viral titers of the rescued viruses were evaluated by plaque assay in MDCK cells. Whereas the wild type virus was rescued (10^−5^ dilution), (B) the mutant virus could not be rescued (“neat” virus, no dilution). (C) Virus titers are presented.

Finally, we investigated the functional impact of M1 arginine triplet mutations by generating a recombinant influenza A(H1N1)pdm09 virus that harbors the R76/77/78A M1 mutation, using a reverse genetic approach. Differently from the wild type virus (Fig 7A), the virus containing the R76/77/78A mutations (Fig 7B) could not be rescued, as shown by virus titration by plaque assay in MDCK cells (Fig 7C). The viral titer was 1.4x10e7 PFU/ml for the wild type virus, whereas no plaque could be detected for the mutant (neat virus). This result indicates that in the influenza A(H1N1)pdm09 strain, the M1 R76/77/78 motif is essential for virus growth because introduction of mutations in this motif is lethal for this virus.

In conclusion, in our experimental system, we could produce VLPs by expressing a minimum set of viral proteins (M1, M2 and NS1/NEP) in HEK 293T cells. We then showed that the M1 R76/77/78A motif is essential for M1 incorporation in VLPs, but not for VLP formation, and is required for influenza A(H1N1)pdm09 virus growth.

## 4-Discussion

In this study, we used a minimal influenza A(H1N1)pdm09 protein expression system in mammalian cells to investigate M1 interaction with cell membranes and trafficking. Our results suggest that M2 and NS1/NEP are the minimum viral determinants for M1 membrane localization in transfected cells. Our findings also show that the M1 R76/77/78 motif is required for M1 binding to model and cell membranes and for influenza A(H1N1)pdm09 virus infectivity.

Our results show that the detection of M1 bound to cell membranes is promoted by co-expression of NS1/NEP (segment 8) and M2 and it is significantly enhanced by the presence also of segment 7 (M). The viral protein NS1 is a general inhibitor of cellular splicing and specifically of segment 7 splicing [27] [28]. Therefore, NS1 expression in our system should increase M1 production (from unspliced segment 7 mRNA) at the expense of the two spliced products M2 and m3. It was also reported that influenza A virus NS1 can stimulate M1 translation [29]. Accordingly, upon co-expression of NS1/NEP, M1 intracellular expression in the presence of M increased (Fig 6A, “intracellular M1” immunoblot, lane 3 versus lane 2) as well as M1 recruitment or detection at the cell membrane (Fig 4), probably due to the higher cytoplasmic M1 concentration. M1 can also interact with the C-terminal domain of NEP via its NLS motif, allowing M1 to exit the nucleus with the vRNPs [21] [30] [31]. As M1 intracellular concentration increased upon co-expression with segment 8 (NS1 and NEP), NS1 and NEP could act as regulators for M1 expression, as previously suggested [29].

Our results with model membranes suggest that M1 R76/77/78 motif promotes M1 interaction with negatively charged LUVs. Unfortunately, we could not test the contribution of M1 NLS basic motif because of our inability to produce and purify the double R76/77/78A-K101/102A mutant recombinant protein in bacteria or in mammalian cells, probably due to its instability. Nevertheless, we could determine that M1-LUV interaction is not enhanced by the presence of PI(4,5)P2, suggesting that the signal targeting M1 to the plasma membrane (M1 assembling site) is not driven by this specific phospholipid. The influenza A(H1N1)pdm09 M1 protein could thus interact with negative phospholipid-enriched membranes, as it is the case for matrix proteins of other RNA enveloped viruses, such as Ebola virus (filovirus) or Murine Leukemia Virus (MLV) (retrovirus) [14] [32] [33]. Differently from the MLV matrix protein that interacts specifically with PI(4,5)P2 in the presence of PS *in vitro* [33], influenza A virus M1, like the Ebola matrix protein VP40, seems to interact with PS only-enriched model membranes [17] [32]. VP40 penetration into the plasma membrane via hydrophobic residues was reported in the case of VP40 [34], but not for M1, so far.

As hundred M1 molecules are localized underneath the membrane of newly formed viral particles, M1 basic residues could interact with negatively charged phospholipids of the cell plasma membrane inner leaflet where viral assembly occurs. This seems to require also another factor because M1 alone cannot bind to cell membranes efficiently (Figs 3A and 4A). The acidic amino acids of the cytoplasmic tails of other transmembrane viral proteins located at the assembly site, such as HA, NA and M2, could be involved. In our minimal system, we found that M2 overexpression in the presence of NS1/NEP is necessary and sufficient. Indeed, membrane flotation assays indicated that mutations in M1 R76/77/78 or M2 CT (E74EY76; M2-mut2) drastically reduce M1 attachment to cell membranes (Fig 4C), even in the presence of NP, HA and NA (Fig 4D). This is consistent with the results by Wang *et al*. [26] and Chen *et al*. [13] and suggests an interaction between M1 and M2 cytoplasmic tail residues. Conversely, HA and NA are not strictly required for M1 membrane localization (Fig 4D). Other studies have shown that M1 alone can localize at the cell membrane in the absence of HA and NA [5] [35], and was recently confirmed using different microscopy techniques in cells and on *in vitro* model membranes [36]. The quite contradictory results obtained in cells could depend on the techniques used to isolate M1-associated membrane fractions [7] [15] [37] [38]. By using a vaccinia virus/T7 polymerase (vac/T7) expression system, it was shown that M1 is a membrane-localized protein in the late steps of infection and its localization is impaired following mutation of the viral NA envelope protein cytoplasmic tail [38]. Kretzschmar and colleagues found that about 20% of total cellular M1 is associated with membranes when M1 is expressed in cells using the vac/T7 expression system [35]. Furthermore, M2 does not seem to influence M1 membrane localization when M1 and the viral envelope HA/NA proteins are also expressed [38], suggesting that viral membrane proteins have a crucial role in M1 membrane localization or targeting. In contrast, using a plasmid expression system, we found that influenza A/H1N1/pdm09 M2 cytoplasmic tail is essential for M1 membrane localization, but not HA and NA are dispensable (Fig 4D), in agreement with [13] [16] [26].

We then found that any mutation in M1 R76/77/78 motif strongly affects M1 cell membrane localization and attachment, differently from what observed for the M1 NLS K101/102A mutant (Figs 3, 4 and 5). This is in agreement with the work by Thaa *et al*. [18] showing that an M1 NLS mutant remains attached to cell membranes. However, the NLS basic residues can be involved in the nuclear export of vRNP complexes (via interaction with NEP, NP or vRNA), or in M1 oligomerization [21] [39] [40]. In our experimental conditions, the R76/77/78A or the K101/102A mutations could have affected M1 multimerization and consequently its membrane binding. Previous studies reported that i*n vitro*, the M1 87–165 segment can self-associate [39], whereas the M1 NLS mutant 95-KAVKLYRKLKR-101 → 95-AAVALYAALAA-101 [40] loses its oligomerization properties [17]. This suggests that the NLS is involved in M1 oligomerization [41]. Indeed, crystallography analyses of M1 N-terminal domain at neutral or low pH (pH~4.5) [30] [42] [43] highlighted the presence of different monomer-monomer arrangements. The authors proposed that M1 oligomerization flexibility could explain its multiple functions during the viral cycle. When looking at the crystal structure of the N-terminal domain at neutral pH (i.e., the pH condition of virus assembly), the interface between M1 monomers involves helix 6 that contains the K101/102 residues [43]. The authors suggested that at this pH, M1 could be organized in the virions in «monomeric building blocks» that polymerize through electrostatic interactions face-to-back [43]. Our data are in agreement with this hypothesis of M1 monomers that interact through the NLS and with an exposed R76/77/78 motif, thus available for other interactions. Therefore, the NLS motif could be involved in M1 oligomerization, and the arginine triplet in M1 interaction with phospholipids and/or M2 CT at the cell membrane. Based on the work by Chen *et al*. [13] and Wang et al. [26], we hypothesized that the M1 R76/77/78 motif could interact with the M2 E74EY76 cytoplasmic tail motif. Unfortunately, we were unable to characterize M1-M2 interaction by immuno-precipitation experiments or Forster Resonance Energy Transfer (FRET) due to (i) the lack of an antibody against influenza A(H1N1)pdm09 M2; (ii) the higher cytosolic concentration of the M1 R76/77/78A mutant compared with M1 WT, and (iii) the very minor interaction between wild type M1 and M2 detectable in cells by FRET, thus not allowing the quantification of a difference between M1 WT and M1 R76/77/78 (data not shown).

Furthermore, M1 R76/77/78 accumulates in intracellular clusters that are not associated with cellular degradative vesicles, and very little with early or recycling endosomes, or with the cell plasma membrane (Fig 5E and S2 Fig). This result indicates that this M1 Arg mutant is clustered in intracellular aggregates (located in the cytosol and in the nucleus) suggesting M1 mutant misfolding and subsequent intracellular sequestration. More studies would be needed in order to identify the nature of these aggregates.

The type of plasmid-based VLP production system used in our study was previously employed for investigating assembly and budding of another influenza A virus strain [13]. Here we found that co-transfection of segments 4, 5 and 6 (HA, NP and NA) did not increase M1 incorporation in VLPs (Fig 6A, lane 6) or VLP release, in agreement with previous studies [23] [44]. In our minimal system, mutations in the M1 R76/77/78 motif impaired M1 incorporation into VLPs, but not VLP formation *per se*, as revealed by EM analysis of thin cell sections (Fig 6C). In agreement with our study, Das *et al*. reported that M1 R77/78A is not incorporated in virions and, consequently, viral production is abolished in an infectious system using the influenza A/WSN/33(H1N1) strain [22]. Similarly, we found that the R76/77/78A mutation is lethal for the influenza A(H1N1)pdm09 strain (Fig 7). Therefore, we propose that in the influenza A/H1N1/pdm09 strain, M1 R76/77/78 motif is involved in M1 cell membrane attachment that triggers its incorporation into newly formed infectious particles.

In conclusion, the M1 R76/77/78 motif plays a role in stabilizing M1 at the cell plasma membrane by interacting with negatively charged phospholipids, such as PS, and/or with M2 CT, as suggested by [13] [16]. The M1 R76/77/78 motif is a determinant of M1 binding to membranes and M1 incorporation in VLPs and is also required for virus infectivity. As this basic motif in M1 N-terminus is highly conserved among influenza A and B virus strains (76-RRR-78 and 75-KRR-77, respectively) [22], but not in C strains, it could become an interesting target for the development of drugs against influenza virus assembly and replication.

## Supporting Information

S1 Fig HA expression in the cytosol and membrane fractions. Expression of influenza A(H1N1)pdm09 HA viral envelope proteins was checked in the Post-Nuclear Supernatant (PNS, i.e. cytosol+cell membranes) after transfection of HEK 293T cells with the indicated plasmids using western blotting with a human serum obtained using an influenza A(H1N1)pdm09 strain isolated from a vaccinated patient. HA-1 of ~63KDa is indicated.
(TIF)

S2 Fig Analysis of M1 R76/77/78A intracellular localization using vesicular markers. A) Immunofluorescence confocal microscopy images of HEK 293T cells transfected with pcDNA-M1 R76/77/78A), pcDNA-M2, pHW2000-NS1/NEP and M* containing the R76/77/78 mutation, and Rab11-mRFP if any (a). M1 was detected using a primary anti-M1 antibody (in green or red, as indicated) and vesicular markers using primary anti-EEA1, CD63, LC3 or Lamp2 antibodies, as indicated (in green). Transmission images are in grey. Scale bars, 5 μm. B) Quantification of co-localization of the M1 R76/77/78 signal with the indicated vesicle markers (Mander’s overlap coefficients).
(TIF)
