# Supplementary figures and images for: Involvement of an Arginine Triplet in M1 Matrix Protein Interaction with Membranes and in M1 Recruitment into Virus-Like Particles of the Influenza A(H1N1)pdm09 Virus
Source: PLoS One. 2016 Nov 4;11(11):e0165421. doi: 10.1371/journal.pone.0165421 (PMC5096668; doi:10.1371/journal.pone.0165421)

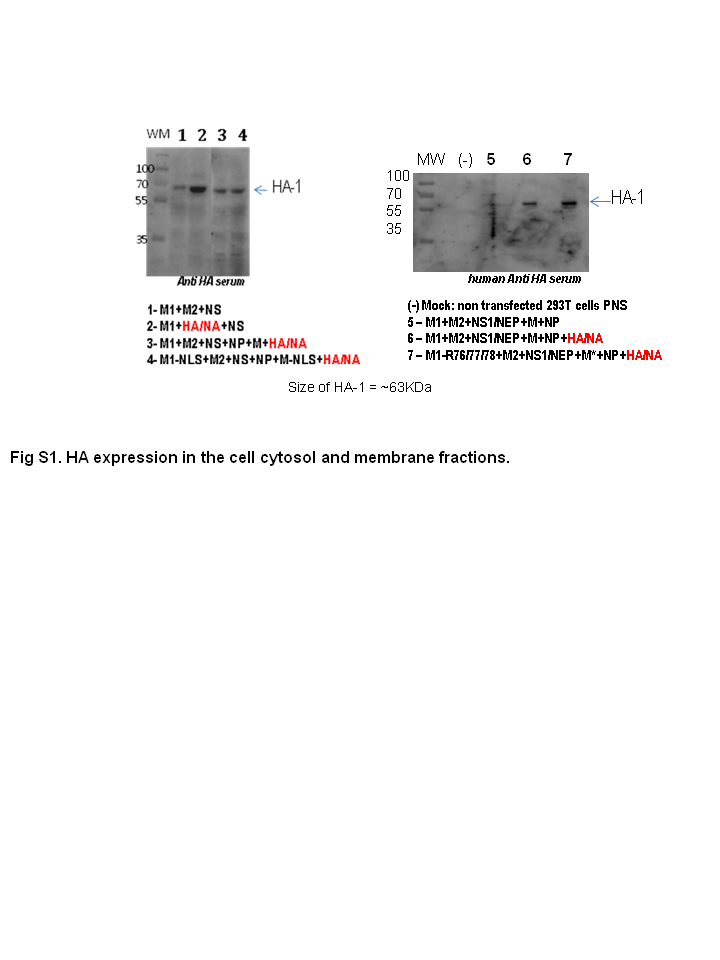

Supplement: S1 Fig — Expression of influenza A(H1N1)pdm09 HA viral envelope proteins was checked in the Post-Nuclear Supernatant (PNS, i.e. cytosol+cell membranes) after transfection of HEK 293T cells with the indicated plasmids using western blotting with a human serum obtained using an influenza A(H1N1)pdm09 strain isolated from a vaccinated patient. HA-1 of ~63KDa is indicated. (TIF) [file pone.0165421.s001.TIF]

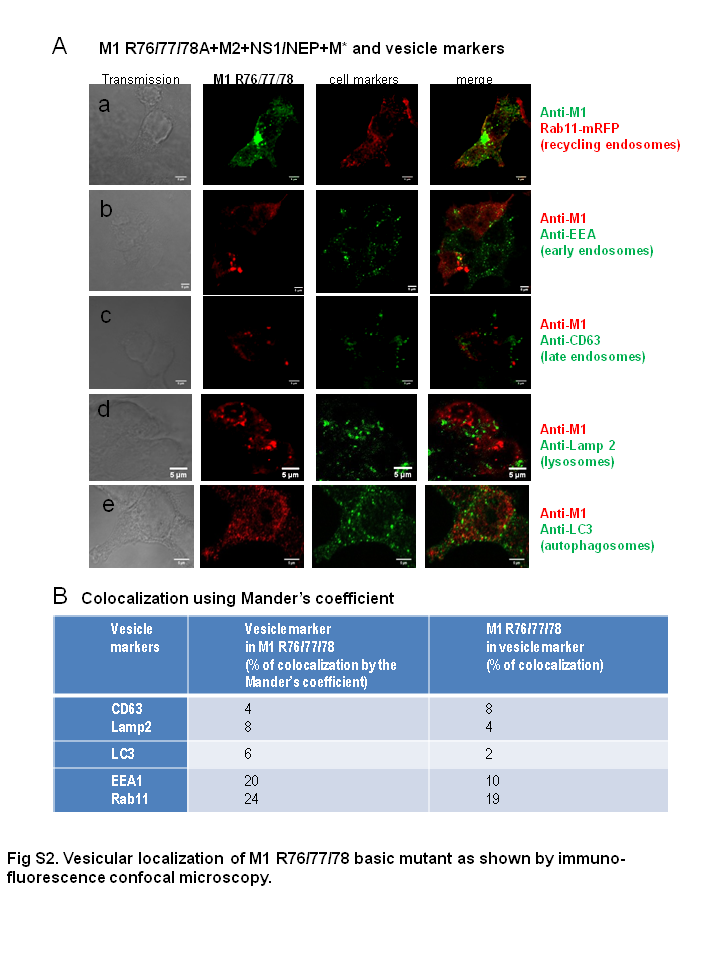

Supplement: S2 Fig — A) Immunofluorescence confocal microscopy images of HEK 293T cells transfected with pcDNA-M1 R76/77/78A), pcDNA-M2, pHW2000-NS1/NEP and M* containing the R76/77/78 mutation, and Rab11-mRFP if any (a). M1 was detected using a primary anti-M1 antibody (in green or red, as indicated) and vesicular markers using primary anti-EEA1, CD63, LC3 or Lamp2 antibodies, as indicated (in green). Transmission images are in grey. Scale bars, 5 μm. B) Quantification of co-localization of the M1 R76/77/78 signal with the indicated vesicle markers (Mander’s overlap coefficients). (TIF) [file pone.0165421.s002.TIF]
